# Supplementary material for: Parabrachial Calca neurons mediate second-order conditioning
Source: Nat Commun. 2024 Nov 9;15:9721. doi: 10.1038/s41467-024-53977-w (PMC11550384; doi:10.1038/s41467-024-53977-w)
Supplement: Supplementary file 1 — Supplementary Information [file 41467_2024_53977_MOESM1_ESM.pdf]

Supplementary Information

Supplementary Figures

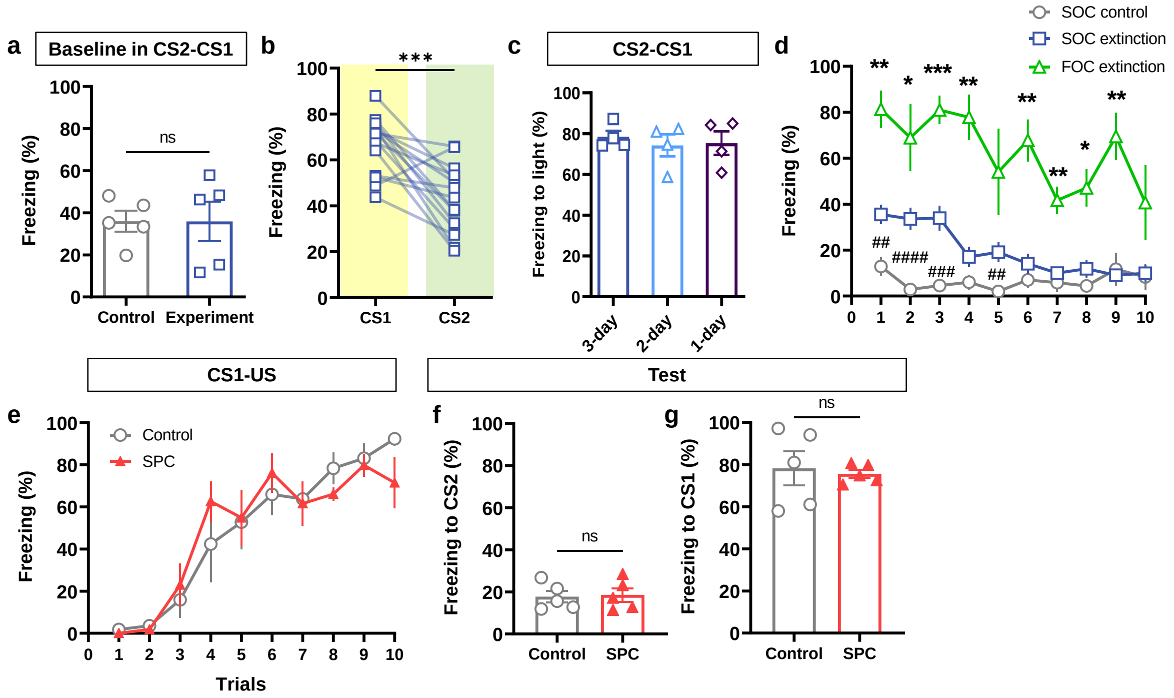

Supplementary Figure 1: Extinction of SOC and sensory preconditioning in mice.

a, Freezing during baseline (1-min before session start) in CS2-CS1 association ($n = 5$ for each group). **b**, Comparison freezing to light CS1 during CS2-CS1 phase and freezing to tone CS2 during test phase ($n = 15$ for experimental animals in Fig. 1). **c**, Freezing response to light CS1 during CS2-CS1 pairing in different training protocol ($n = 4$ for each group). **d**, Freezing level during extinction trials of first-order conditioning (FOC extinction, $n = 4$), second-order conditioning (SOC extinction, $n = 15$), and control (SOC control, $n = 9$). Asterisks (*) indicate comparison between FOC extinction and SOC extinction. Pound signs (#) indicate comparison between SOC extinction and control. **e,** Learning curves during CS1-US association of sensory preconditioning. SPC, sensory preconditioning. **e**, Freezing responses to the tone CS2 on test phase of sensory preconditioning protocol. **f**, Freezing responses to the light CS1 during test phase. **e-g**, $n = 5$ for each group. Data are mean ± SEM, *$p < 0.05$, **$p < 0.01$, ***$p < 0.001$, ##$p < 0.01$, ###$p < 0.001$, ####$p < 0.0001$. Source data are provided as a Source Data file.

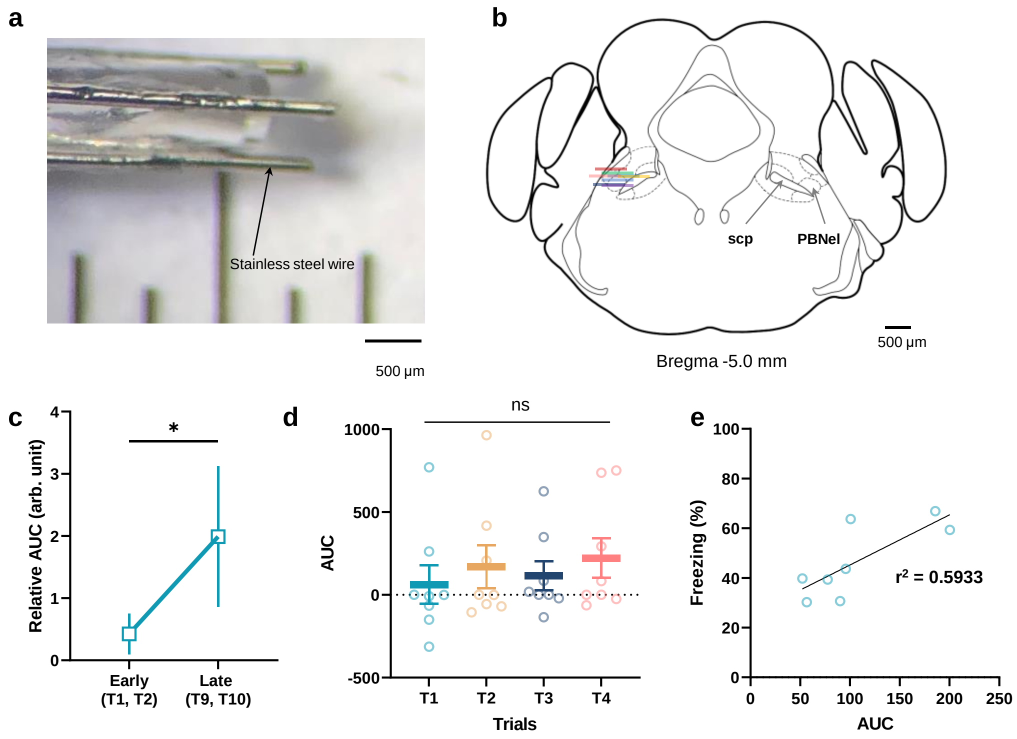

Supplementary Figure 2: GRIN lens implantation in PBN and AUC analysis based on trials.

a, Picture of prong wires attached to GRIN lens for minimizing motion artifact. **b**, Schematic depiction of GRIN lens placements over PBN. Note that PBN section is adapted from Franklin and Paxinos mouse brain atlas[1]. **c**, AUC of $CGRP^{PBN}$ neurons during CS1 across early and late trials during CS1-US association (103 neurons). **d**, AUC of $CGRP^{PBN}$ neurons during tone CS2 across the trials in CS2-CS1 association ($n = 8$). **e**, Linear regression between AUC during tone CS2 in test day and freezing behavior ($n = 8$). The equation of line graph is as $Y = 0.2018X + 25.04$. The data indicate individual animals; plotted as mean ± SEM. Source data are provided as a Source Data file.

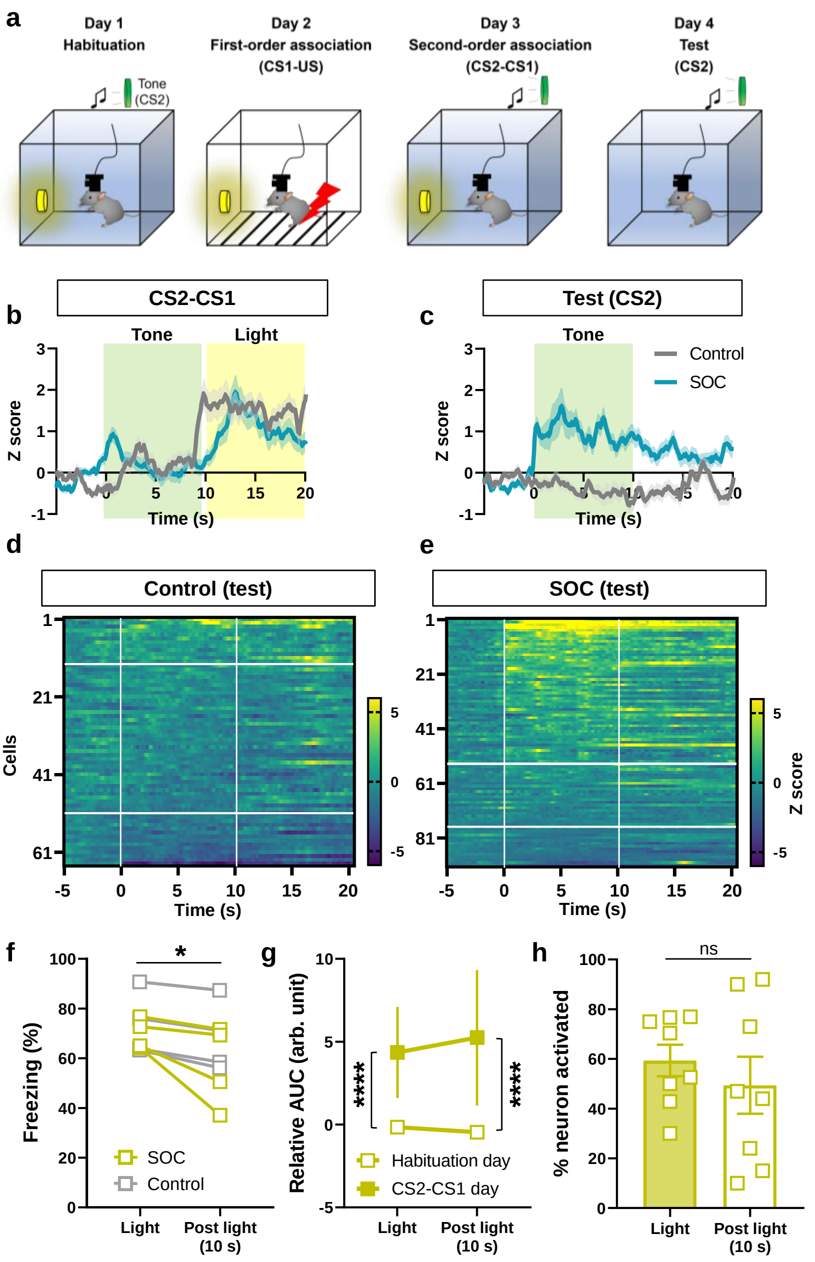

Supplementary Figure 3: CGRP^{PBN} neuronal activity during SOC in the protocol designed to minimize contextual contribution.

a, Schematic of the experimental paradigm of SOC minimizing contextual contribution. **b,** Average traces of CGRP^{PBN} neurons in the control and the SOC group in response to tone CS2 and light CS1 during the CS2-CS1 association and **c,** test days. **d,** Heat map showing individual neuronal responses of the control group to tone CS2 on the test day. **e,** Heat map showing individual neuronal responses of the experimental group to tone CS2 on the test day. **b-e,** *n* = 64 from 4 animals for control group and *n* = 91 neurons from 4 animals for SOC group. **f,** Percent of time freezing during light CS1 on the CS2-CS1 association day (*n* = 8). **g,** AUC during light CS1 and post-light (10 s), compared to the pre 10 s of baseline. Open dots indicate responses of the CGRP^{PBN} neurons to the light CS1 during habituation day

(108 neurons). Closed dots indicate responses of the CGRP^{PBN} neurons to the light CS1 during CS2-CS1 association day (155 neurons) **h,** Percentage of increased responses of CGRP^{PBN} neurons during light CS1 and post-light ($n = 8$). Dots represent individual animals. The data are plotted as mean ± SEM. *$p < 0.05$. Source data are provided as a Source Data file.

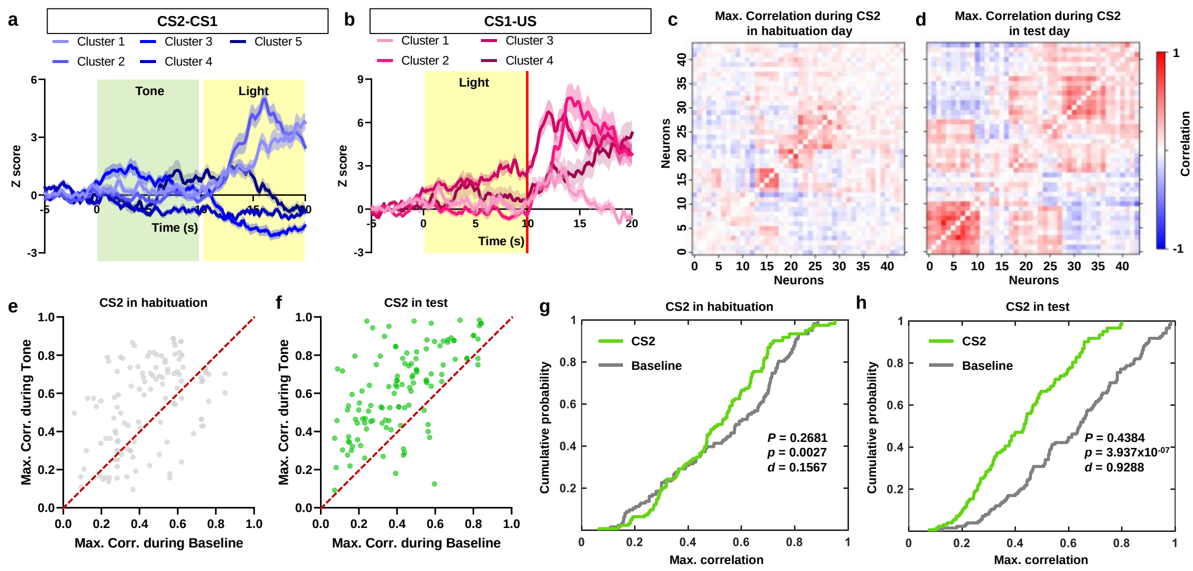

Supplementary Figure 4: Cluster and correlation analysis of CGRP^{PBN} neurons during SOC.

a, Clustered activities of CGRP^{PBN} neurons during CS2-CS1 association (197 neurons). **b**, Clustered activities of CGRP^{PBN} neurons during CS1-US association (103 neurons). **c**, Heatmap of maximum correlations within CGRP^{PBN} neurons from representative animal before SOC (habituation). **d**, Heatmap of maximum correlations within CGRP^{PBN} neurons from representative animal after SOC (test). **e**, Linear regression of maximum correlations of CGRP^{PBN} neurons in habituation phase. **f**, Linear regression of maximum correlations of CGRP^{PBN} neurons in test day**.** **g**, Cumulative distribution function of maximum correlations during tone CS2 and baseline in habituation day. Note that baseline indicates neural activities of first 5 min in test chamber before session start. P indicates pairwise Pearson's correlation. *p* indicates paired t-test. *d* indicates Cohen's *d* which represents effect size. **h**, Cumulative distribution function of mean correlations during tone CS2 and baseline in test day. **e-h,** *n* = 122 neurons. Data are mean ± SEM. Dots indicate individual neurons. Source data are provided as a Source Data file.

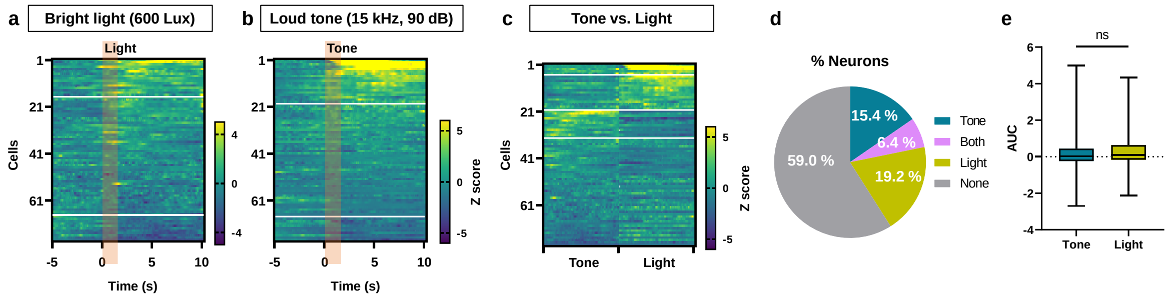

Supplementary Figure 5: CGRP^{PBN} neuronal responses to the noxious light and tone before learning.

a, Heat map showing individual CGRP^{PBN} neuron responses to bright light (1 s, 600 lux) and **b,** loud tone (1 s, 15 kHz, 90 dB). Neurons are aligned based on activity between 0 and 10 s. The top neurons (above the first white line) show increased activity, the middle neurons are non-responsive, and the bottom neurons (below the second white line) show decreased activity. **c,** Comparison of responses between the loud tone and bright light based on activity patterns. From top to bottom: neurons activated by both stimuli, neurons activated only by bright light, neurons activated only by loud tone, and neurons non-responsive to both stimuli. **d,** Proportion of CGRP^{PBN} neurons based on their responses. **e,** Average AUC of neurons 10 s after the onset of the loud tone or bright light. **a-e,** $n = 78$ neurons from 4 animals. The boxes indicate first and third quartile; line in the box plot indicate median; whiskers indicate 5-95 percentile of population. Source data are provided as a Source Data file.

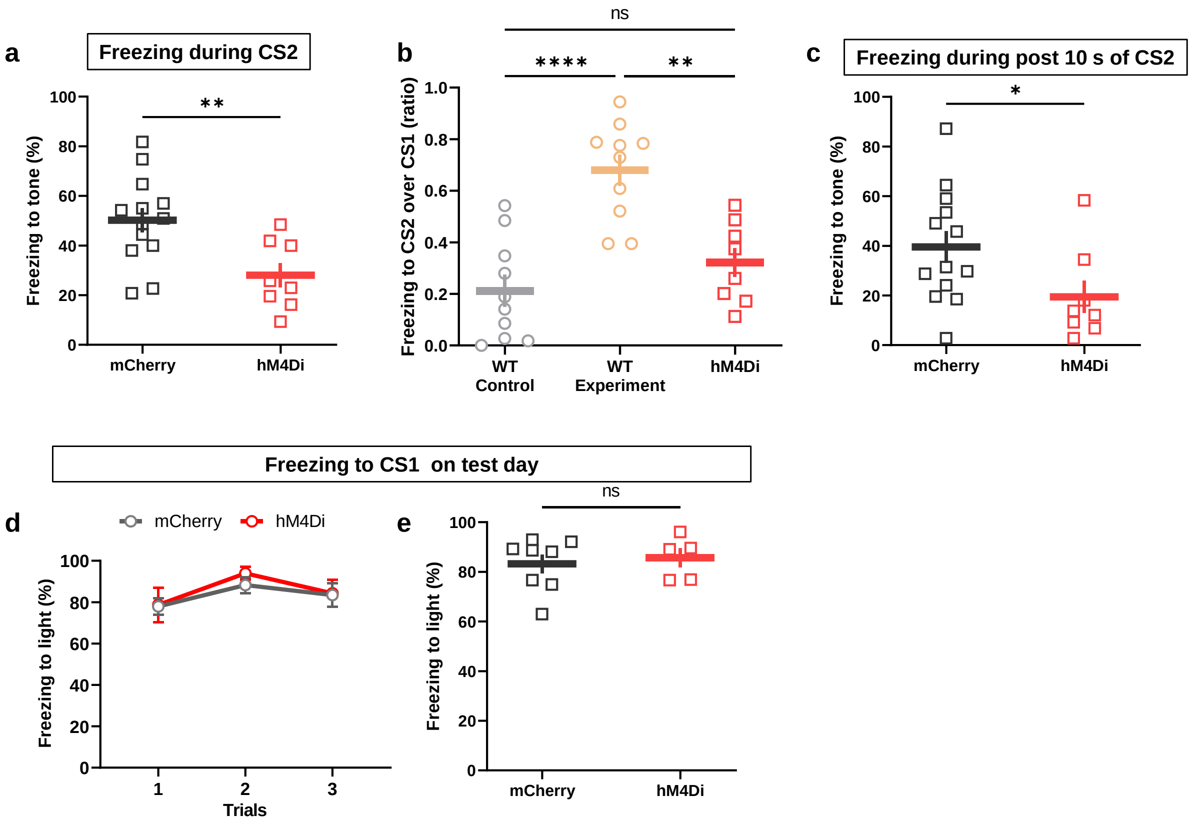

Supplementary Figure 6: Transient inhibition of $CGRP^{PBN}$ neurons during second-order association attenuates freezing to CS2 and post CS2. a, Freezing responses to tone CS2 of control (*n* = 13) and hM4Di (*n* = 8) groups after SOC. **b**, Comparison of freezing ratio CS2 over CS1 between WT mice (*n* = 10) experiments (*n* = 10) and hM4Di (*n* = 8) group. Note that WT control did not receive second-order association. **c**, Freezing responses to post CS2 (10 sec after CS2) of control (*n* = 13) and hM4Di (*n* = 8) groups in test phase. **d,** Freezing responses to CS1 of control (*n* = 8) and hM4Di (*n* = 5) groups in test phase based on trials. **e,** Average of trials in **d.** Data are plotted as mean ± SEM, *p < 0.05, **p < 0.01, ****p < 0.0001. Source data are provided as a Source Data file.

Summary of the statistical analysis

Figure	Mean<br>(number of data)	Normality test<br>(Shapiro-Wilk test, P>0.05; passed)	Statistical test	Comparison	Results (P value)
**Fig. 1c**	Experiment CS1 = 72.71 (5)<br>Experiment CS2 = 53.23 (5)<br>Control CS1 = 77.77 (5)	Experiment CS1, P = 0.3296<br>Experiment CS2, P = 0.3635<br>Control CS1, P = 0.2670	Two-way RM ANOVA<br>F (1, 8) = 86.61	Experiment CS1 vs. Experiment CS2	< 0.0001
			Two-way ANOVA<br>F (1, 8) = 43.17	Control CS1 vs. Experiment CS2	0.0002
**Fig. 1d**	Control = 7.143 (14)<br>Experiment = 42.81 (15)	P = 0.0795 for Control<br>P = 0.6454 for Experiment	Unpaired t-test (Two-tailed)<br>t = 8.381, df = 27	Control vs. Experiment	<0.0001
**Fig. 1e**	Median difference of Experiment= 19.72 (10)	10 kHz, P = 0.0200<br>5 kHz, P = 0.3954	Wilcoxon signed rank test<br>W = 10	10 kHz vs 5 kHz	0.0020
**Fig. 1f**	Control = 17.82 (11)<br>Experiment = 48.45 (11)<br>30-s interval = 11.84 (12)<br>Reversed = 16.60 (13)<br>Simultaneous = 14.36 (10)	Control, P = 0.0229<br>Experiment, P = 0.0239<br>30-s interval, P = 0.0577<br>Reversed, P = 0.1172<br>Simultaneous, P = 0.3331	Kruskal-Wallis test<br>K = 21.06<br>P = 0.0003	Experiment vs. Control	<0.0001
				Experiment vs. 30-s interval	0.0052
				Experiment vs. Reversed	0.0079
				Experiment vs. Simultaneous	0.0058
**Fig. 1g**	Control = 7.84 (5)<br>Experiment = 42.53 (5)<br>2-SOC = 45.58 (5)	Control, P = 0.5247<br>Experiment, P = 0.6563<br>2-SOC, P = 0.4133	One-way ANOVA<br>F (2, 12) = 2.245<br>P = 0.0017	Control vs. Experiment	0.0052
				Control vs. 2-SOC	0.0028
				Experiment vs. 2-SOC	0.9366
**Fig. 1i**	3-day = 33.78 (9)<br>2-day = 7.852 (10)<br>1-day = 6.370 (10)<br>Control = 6.370 (5)	3-day, P = 0.3057<br>2-day, P = 0.8658<br>1-day, P = 0.4034<br>Control, P = 0.2580	One-way ANOVA<br>F = 19.85<br>P < 0.0001	3-day vs. 2-day	< 0.0001
				3-day vs. 1-day	< 0.0001
				3-day vs. Control	< 0.0001
**Fig. 2d**	Baseline = 23.76 (8)<br>Tone = 45.49 (8)<br>Post tone = 42.86 (8)	Baseline, P = 0.1290<br>Tone, P = 0.6057<br>Post tone, P = 0.9724	One-way RM ANOVA<br>F (1.810, 12.67) = 7.242<br>P = 0.0092	Baseline vs. Tone	0.0464
				Baseline vs. Post tone	0.0663
				Tone vs Post tone	0.9473
**Fig. 3a (left)**	Habituation = 13.81 (199)<br>CS1-US = 74.59 (103)<br>CS2-CS1 = 74.59 (197)	Habituation, P < 0.0001<br>CS1-US, P = 0.0016<br>CS2-CS1, P < 0.0001	Kruskal-Wallis test<br>K = 15.15<br>P = 0.0005	Habituation vs. CS1-US	0.0009
				Habituation vs. CS2-CS1	0.0154
				CS1-US vs. CS2-CS1	0.9971

**Fig. 3a (right)**	Habituation = -3.068 (199)<br>CS2-CS1 = 13.17 (197)<br>Test = 110.2 (209)	Habituation, P < 0.0001<br>CS1-CS2, P = 0.0045<br>Test, P < 0.0001	Kruskal-Wallis test<br>K = 74.23<br>P < 0.0001	Habituation vs. CS2-CS1	0.2775
				Habituation vs. Test	< 0.0001
				CS2-CS1 vs. Test	< 0.0001
**Fig. 3e**	Habituation = 155.5 (18)<br>CS2-CS1 = 25.39 (18)<br>Test = 81.08 (18)	Habituation, P = 0.2309<br>CS2-CS1, P = 0.4220<br>Test, P = 0.1555	One-way RM ANOVA<br>F (1.833, 31.15) = 12.15<br>P = 0.0002	Habituation vs. CS2-CS	0.0013
				Habituation vs. Test	0.0232
				CS2-CS1vs. Test	0.0749
**Fig. 3f**	Habituation = -2.388 (76)<br>CS2-CS1 = -4.382 (76)<br>Test = 55.77 (76)	Habituation, P = 0.0593<br>CS2-CS1, P = 0.0086<br>Test, P = 0.0734	Friedman test<br>F = 19.13<br>P<0.0001	Habituation vs. CS2-CS1	>0.9999
				Habituation vs. Test	0.0011
				CS2-CS1 vs. Test	0.0002
**Fig. 3g**	Habituation = -135.4 (28)<br>CS2-CS1 = 32.57 (28)<br>Test = 95.70 (28)	Habituation, P = 0.9992<br>CS2-CS1, P = 0.3078<br>Test, P = 0.0027	Friedman test<br>F = 35.64<br>P<0.0001	Habituation vs. CS2-CS1	<0.0001
				Habituation vs. Test	<0.0001
				CS2-CS1 vs. Test	0.4247
**Fig. 3j**	Mean difference = 309.9 in 1st-order<br>-10.86 in 2nd-order<br>CS1/CS2 (25)<br>CS2 (28)	CS1/CS2, P = 0.0730<br>CS2, P = 0.4432	Two-way RM ANOVA<br>F (1, 51) = 72.80<br>P<0.0001	CS1/CS2 vs. CS2 in 1st-order	<0.0001
				CS1/CS2 vs. CS2 in 2nd-order	0.9087
**Fig. 3i**	Mean difference = -10.86 in 0-10 s<br>-90.49 in 10-20 s<br>CS1/CS2 (25)<br>CS2 (28)	CS1/CS2, P = 0.2738<br>CS2, P < 0.0001	Two-way RM ANOVA<br>F (1, 51) = 4.049<br>P=0.0495	CS1/CS2 vs. CS2 in 0-10 s	0.9451
				CS1/CS2 vs. CS2 10-20 s	0.0286
**Fig. 4c**	mCherry light = 83.88 (13)<br>hM4Di light = 88.64 (8)<br>mCherry tone = 56.13 (13)<br>hM4Di tone = 54.02 (8)	mCherry light, P = 0.5215<br>hM4Di light, P = 0.7614<br>mCherry tone, P = 0.3436<br>hM4Di tone, P = 0.4700	Two-way ANOVA<br>F (3,57) = 1.757<br>P = 0.1656	mCherry light vs. hM4Di light	0.3958
			Two-way ANOVA<br>F (3, 57) = 0.2211<br>P = 0.8813	mCherry tone vs. hM4Di tone	0.68347
**Fig. 4d**	mCherry = 0.6133 (13)<br>hM4Di = 0.3221 (8)	mCherry, P = 0.5845<br>hM4Di, P = 0.6684	Unpaired t-test (Two-tailed)<br>t = 3.038, df = 19	mCherry vs. hM4Di	0.0068
**Supplementary Fig. 1a**	Control = 36.00 (5)<br>Experiment = 35.89 (5)	Control, P = 0.7714<br>Experiment, P = 0.2014	Unpaired t-test (Two-tailed)<br>t = 0.0100, df = 8	Control vs. Experiment	0.9922
**Supplementary Fig. 1c**	3-day = 78.42<br>2-day = 74.19<br>1-day = 75.35	3-day, P = 0.1183<br>2-day, P = 0.2202<br>1-day, P = 0.3445	One-way ANOVA<br>F (2, 9) = 0.1997<br>P = 0.8225	3-day vs. 2-day	0.7716

**Supplementary Fig. 1d**	SOC control = 12.89, 2.85, 4.52, 6.04, 2.00, 7.00, 5.85, 4.41, 11.70, 8.26 (9)<br>SOC extinction = 35.53, 33.56, 33.91, 17.02, 19.11, 14.09, 9.98, 11.91, 9.09, 9.89 (15)<br>FOC extinction = 81.33, 68,92, 81,00, 77,83, 54,00, 67,57, 41,67, 47.08, 69.60, 40.67 (4)	SOC control, P = 0.8663<br>SOC extinction, P = 0.9357<br>FOC extinction, P = 0.8853	Two-way RM ANOVA<br>F (18, 225) = 3.007<br>P < 0.001	3-day vs. 1-day	0.8697
				SOC extinction vs. FOC extinction	0.0023, 0.0394, 0.0005, 0.0070, 0.1341, 0.0081, 0.0062, 0.0224, 0.0088, 0.2664
**Supplementary Fig. 1e**	Control = 50.00 (5)<br>SPC = 49.73 (5)	Control, P = 0.3065<br>SPC, P = 0.0289	Two-way RM ANOVA<br>F (9, 72) = 1.033<br>P = 0.4230	Control vs. SPC	0.9697
**Supplementary Fig. 1f**	Control = 17.71 (5)<br>SPC = 18.56 (5)	Control, P = 0.4262<br>SPC, P = 0.6146	Unpaired t-test (Two-tailed)<br>t = 0.1974, df = 8	Control vs. SPC	0.8485
**Supplementary Fig. 1g**	Control = 78.29 (5)<br>SPC = 75.62 (5)	Control, P = 0.2897<br>SPC, P = 0.4856	Unpaired t-test (Two-tailed)<br>t = 0.3184, df = 8	Control vs. SPC	0.7584
**Supplementary Fig. 2c**	Median difference = 0.1786 (103 neurons)	Early, P < 0.0001<br>Late, P < 0.0001	Wilcoxon signed rank test<br>W = 1196	Early vs. Late	0.0490
**Supplementary Fig. 2d**	T1 = 61.75 (8)<br>T2 = 169.6 (8)<br>T3 = 115.0 (8)<br>T4 = 221.8 (8)	T1, P = 0.0788<br>T2, P = 0.0128<br>T3, P = 0.0388<br>T4, P = 0.0134	Friedman test<br>F = 1.650<br>P = 0.6481	T1vs. T2	>0.9999
				T1 vs. T3	>0.9999
				T1 vs. T4	>0.9999
				T2 vs. T3	>0.9999
				T2 vs. T4	>0.9999
				T3 vs. T4	>0.9999
**Supplementary Fig. 2e**	AUC = 107.34 (8)<br>Freezing = 45.49 (8)	AUC, P = 0.2037<br>Freezing, P = 0.0678	Simple linear regression<br>F (1, 6) = 8.751<br>P = 0.0253	Equation<br>Y = 0.2018X + 25.04	r^2 = 0.5933
**Supplementary Fig. 3f**	Mean difference = -8.833 (total 8, control and experiment)	Light, P = 0.0765<br>Post light, P = 0.9358	Paired t-test (Two-tailed)<br>t = 3.043, df = 7	Light vs. Post light	0.0188
**Supplementary Fig. 3g**	Median difference = -0.2432 (155 neurons)	Light (SOC), P < 0.001<br>Post light (SOC), P < 0.001	Wilcoxon signed rank test<br>W = -2670	Light (SOC) vs. Post light (SOC)	0.0168
	Median difference = -0.0746 (108 neurons)	Light (Habituation), P < 0.001<br>Post light (Habituation), P < 0.001	Wilcoxon signed rank test<br>W = -1252	Light (Habituation) vs. Post light (Habituation)	0.0550
	Median difference = -0.9825	Light (SOC), P < 0.001<br>Light (Habituation), P < 0.001	Mann Whitney test<br>U = 4195	Light (Habituation) vs. Light (SOC)	<0.0001
	Median difference = -0.6531	Post light (SOC), P < 0.001	Mann Whitney test	Post light (Habituation)	<0.0001

		Post light (Habituation), P < 0.001	U = 5601	vs. Post light (SOC)	
**Supplementary Fig. 3h**	Mean differences = -9.931 (8)	Light, P = 0.1978<br>Post light, P = 0.3281	Paired t-test (Two-tailed)<br>t = 1.245, df = 7	Light vs. Post light	0.2534
**Supplementary Fig. 4g**	Baseline = 0.443 (122)<br>CS2 = 0.540 (122)	Baseline, P = 0.0222<br>CS2, P < 0.0001	Pairwise Pearson's correlation<br>P = 0.2681	Baseline vs. CS2	P = 0.0027<br>Cohen's d = 0.1567
**Supplementary Fig. 4h**	Baseline = 0.421 (122)<br>CS2 = 0.618 (122)	Baseline, P = 0.0046<br>CS2, P = 0.0124	Pairwise Pearson's correlation<br>P = 0.4384	Baseline vs. CS2	P = $3.937*10^{-7}$<br>Cohen's d = 0.9288
**Supplementary Fig. 5e**	Median differences = 0.03921	Tone, P < 0.0001<br>Light, P < 0.0001	Wilcoxon signed rank test<br>W = 365.0	Tone vs. Light	0.3667
**Supplementary Fig. 6a**	mCherry = 50.24 (13)<br>hM4Di = 28.03 (8)	mCherry, P = 0.9041<br>hM4Di, P = 0.6037	Unpaired t-test (Two-tailed)<br>t = 3.001, df = 19	mCherry vs. hM4Di	0.0073
**Supplementary Fig. 6b**	WT control = 0.2114 (10)<br>WT experiment = 0.6799 (10)<br>hM4Di = 0.3221 (8)	WT control, P = 0.2596<br>WT experiment, P = 0.3165<br>hM4Di = 0.6684	One-way ANOVA<br>F (2, 25) = 17.48<br>P < 0.0001	hM4Di vs. WT control	0.4269
				hM4Di vs. WT experiment	0.0011
				WT control vs. WT experiment	<0.0001
**Supplementary Fig. 6c**	mCherry = 39.54 (13)<br>hM4Di = 19.43 (8)	mCherry, P = 0.8760<br>hM4Di, P = 0.0439	Mann Whitney test<br>U = 22	mCherry vs hM4Di	0.0302
**Supplementary Fig. 6d**	mCherry = 83.21 (8)<br>hM4Di = 85.67 (5)	mCherry, P = 0.0938<br>hM4Di, P = 0.2596	Two-way RM ANOVA<br>F (2, 22) = 0.1924<br>P = 0.8264	mCherry vs hM4Di	T1, 0.9995
					T2, 0.8464
					T3, 0.9992
**Supplementary Fig. 6e**	mCherry = 83.21 (8)<br>hM4Di = 85.67 (5)	mCherry, P = 0.0938<br>hM4Di, P = 0.2596	Unpaired t-test<br>t = 0.435, df = 11	mCherry vs hM4Di	0.6718

Reference

1. Franklin, K. and Paxinos G. *et al.* Paxinos and Franklin's the Mouse Brain in Stereotaxic Coordinates; 5th edition. *Academic Press*, (2019).
