# Supplementary material for: Parabrachial Calca neurons mediate second-order conditioning
Source: Nat Commun. 2024 Nov 9;15:9721. doi: 10.1038/s41467-024-53977-w (PMC11550384; doi:10.1038/s41467-024-53977-w)
Supplement: Supplementary file 3 — Reporting Summary [file 41467_2024_53977_MOESM3_ESM.pdf]

nature portfolio

Corresponding author(s): Richard D. Palmiter

Last updated by author(s): Oct 2, 2024

Reporting Summary

Nature Portfolio wishes to improve the reproducibility of the work that we publish. This form provides structure for consistency and transparency in reporting. For further information on Nature Portfolio policies, see our Editorial Policies and the Editorial Policy Checklist.

Statistics

For all statistical analyses, confirm that the following items are present in the figure legend, table legend, main text, or Methods section.

n/a	Confirmed	
☐	☒	The exact sample size (*n*) for each experimental group/condition, given as a discrete number and unit of measurement
☐	☒	A statement on whether measurements were taken from distinct samples or whether the same sample was measured repeatedly
☐	☒	The statistical test(s) used AND whether they are one- or two-sided<br>*Only common tests should be described solely by name; describe more complex techniques in the Methods section.*
☐	☒	A description of all covariates tested
☐	☒	A description of any assumptions or corrections, such as tests of normality and adjustment for multiple comparisons
☐	☒	A full description of the statistical parameters including central tendency (e.g. means) or other basic estimates (e.g. regression coefficient) AND variation (e.g. standard deviation) or associated estimates of uncertainty (e.g. confidence intervals)
☐	☒	For null hypothesis testing, the test statistic (e.g. F, t, r) with confidence intervals, effect sizes, degrees of freedom and P value noted<br>*Give P values as exact values whenever suitable.*
☒	☐	For Bayesian analysis, information on the choice of priors and Markov chain Monte Carlo settings
☒	☐	For hierarchical and complex designs, identification of the appropriate level for tests and full reporting of outcomes
☐	☒	Estimates of effect sizes (e.g. Cohen's *d*, Pearson's *r*), indicating how they were calculated

Our web collection on statistics for biologists contains articles on many of the points above.

Software and code

Policy information about availability of computer code

Data collection	1. IDAS 2.2.0 and nVista (Inscopix) for Ca2+ imaging data acquisition. 2. MED-PC IV 4.2 (Med Associate) for running behavioral experiment. 3. BZ-X viewer BZ-X700 (Keyence) for histology data acquisition.
Data analysis	1. IDPS 1.9.1 and IDEAS 23.7 (Inscopix) for Ca2+ imaging data analysis. 2. Custimized MATLAB R2022b code for data sorting. 3. Ethovision XT 15 (Noldus) for video analysis. 4. BZ-X analyzer (Keyence) for histology analysis. 5. GraphPad Prism 9 for graph generation and statistical anlaysis.

For manuscripts utilizing custom algorithms or software that are central to the research but not yet described in published literature, software must be made available to editors and reviewers. We strongly encourage code deposition in a community repository (e.g. GitHub). See the Nature Portfolio guidelines for submitting code & software for further information.

Data

Policy information about availability of data

All manuscripts must include a data availability statement. This statement should provide the following information, where applicable:

- Accession codes, unique identifiers, or web links for publicly available datasets
- A description of any restrictions on data availability
- For clinical datasets or third party data, please ensure that the statement adheres to our policy

Source data are provided with this paper. The customized code is available in GitHub (https://doi.org/10.5281/zenodo.10725342).

Research involving human participants, their data, or biological material

Policy information about studies with human participants or human data. See also policy information about sex, gender (identity/presentation), and sexual orientation and race, ethnicity and racism.

Reporting on sex and gender	n/a
Reporting on race, ethnicity, or other socially relevant groupings	n/a
Population characteristics	n/a
Recruitment	n/a
Ethics oversight	n/a

Note that full information on the approval of the study protocol must also be provided in the manuscript.

Field-specific reporting

Please select the one below that is the best fit for your research. If you are not sure, read the appropriate sections before making your selection.

☒ Life sciences ☐ Behavioural & social sciences ☐ Ecological, evolutionary & environmental sciences

For a reference copy of the document with all sections, see nature.com/documents/nr-reporting-summary-flat.pdf

Life sciences study design

All studies must disclose on these points even when the disclosure is negative.

Sample size	An online power and sample size calculator was used to determine an effective sample size for statistical comparisons (http://powerandsamplesize.com)
Data exclusions	The extent of viral transduction in both left and right PBN was determined histologically and only those mice with ample transduction on both sides (judged by someone blind to the experiment) were included in the analysis. For Ca2+ imaging, only mice with >15 neurons in the field of view were used for experiments.
Replication	The experiments performed with multiple animals to ensure reproducibility. All number of replications (subjects) is noted in the figure legends. For reproducibility, we included detailed protocol and sources of all reagents in the manuscript.
Randomization	Animals from the same litter were split randomly between control and experimental groups, with an nearly equal number of male and female mice in each group.
Blinding	Investigators were blinded to the group allocation during data collection and analysis of behavioral experiments. Ca2+ imaging and its analysis were not performed under blinded conditions due to the use of a single group.

Reporting for specific materials, systems and methods

We require information from authors about some types of materials, experimental systems and methods used in many studies. Here, indicate whether each material, system or method listed is relevant to your study. If you are not sure if a list item applies to your research, read the appropriate section before selecting a response.

Materials & experimental systems

n/a	Involved in the study	
☐	☒	Antibodies
☒	☐	Eukaryotic cell lines
☒	☐	Palaeontology and archaeology
☐	☒	Animals and other organisms
☒	☐	Clinical data
☒	☐	Dual use research of concern
☒	☐	Plants

Methods

n/a	Involved in the study	
☒	☐	ChIP-seq
☒	☐	Flow cytometry
☒	☐	MRI-based neuroimaging

Antibodies

Antibodies used	1. Chicken-anti-GFP (Abcam, ab13970). 2. Rabbit-anti-DsRed (Takara, 632496). 3. Alexa Fluor 488 donkey anti-chicken (Jackson ImmunoResearch, #2340375). 4. Alexa Fluor 594 donkey anti-rabbit (Jackson ImmunoResearch, #2340621).
Validation	Antibodies were validated by the manufacturer for immunofluorescece use in mouse tissue.<br>1. Chicken-anti-GFP (Abcam, ab13970). https://www.abcam.com/products/primary-antibodies/gfp-antibody-ab13970.html?productWallTab=ShowAll<br>2. Rabbit-anti-DsRed (Takara, 632496). https://www.takarabio.com/products/antibodies-and-elisa/fluorescent-protein-antibodies/red-fluorescent-protein-antibodies<br>3. Alexa Fluor 488 donkey anti-chicken (Jackson ImmunoResearch, #2340375). https://www.jacksonimmuno.com/catalog/products/703-545-155<br>4. Alexa Fluor 594 donkey anti-rabbit (Jackson ImmunoResearch, #2340621). https://www.jacksonimmuno.com/catalog/products/711-585-152

Animals and other research organisms

Policy information about studies involving animals; ARRIVE guidelines recommended for reporting animal research, and Sex and Gender in Research

Laboratory animals	The WT mice (C57BL/6J, Jackson Lab #000664), CalcaCre/+ (Jackson Lab #33168), CalcafrtCre/+ (Palmiter Lab) and Ai162 (Jackson Lab #031562) were used in this study. The CalcaCre/+ mice were backcrossed to C57BL/6J mice for >10 generations. Mice were housed on a 12-h light and dark cycle at ~22C with food and water available ad libitum.
Wild animals	This study did not involve wild animals.
Reporting on sex	Male and female mice were used in the study. Nearly equal number of male and female mice were used in each group.
Field-collected samples	This study did not involve field-collected samples.
Ethics oversight	All experiments were approved by the University of Washington Institutional Animal Care and Use Committee and were performed under the guidelines described in the US National Institutes of Health Guide for the Care and Use of Laboratory Animals.

Note that full information on the approval of the study protocol must also be provided in the manuscript.

Plants

Seed stocks	n/a
Novel plant genotypes	n/a
Authentication	n/a
